# An Exploration of Heart Rate Response and Blood Tetrahydrocannabinol (THC) Levels to Commercially Available Cannabis Edibles by Dose

**DOI:** 10.21203/rs.3.rs-9762624/v1

**Published:** 2026-06-11

**Authors:** Sarah Limbacher, Sarah Bird, Julia Wrobel, Sparsh Jain, Ashley Brooks-Russell

**Affiliations:** Colorado School of Public Health; Colorado School of Public Health; Emory University; Transportation Research Center, Inc.; Colorado School of Public Health

## Abstract

**Introduction:**

Studies have shown that tetrahydrocannabinol (THC) consumption can acutely increase heart rate (HR) regardless of the percent concentration of the product consumed, mode of consumption (e.g. flower, edibles, concentrate), and tolerance. Prior studies have found that the magnitude of this effect is slightly larger in inhaled products, however few purposeful studies on HR response to commercially available edible cannabis have been done.

**Materials & Methods:**

Participants (N = 59) were consented and asked to use at least 5mg of cannabis edibles during their study visit. Participants were enrolled into one of two cannabis use categories based on self-reported frequency of their cannabis use. Participants who use any cannabis product daily (defined for study purposes as 29 days of the last 30) and occasionally (participants who use cannabis at least one time per month but no more than three times per week). Venous blood samples were taken at baseline and after edible consumption, from an intravenous line at an average of 43 (post 1), 89 (post 2) and 178 minutes (post 3) post cannabis use. Participants were seated for all HR collection which occurred at baseline and an average of 39 (post 1), 84 (post 2), and 163 (post3) minutes after the participant’s end of cannabis consumption. Medications and medical conditions that suppress HR or induce elevated HR were identified and controlled for in this analysis.

**Results:**

Overall, we observed a statistically significant but small non-clinically significant decrease in HR after consumption of cannabis edible. This was observed across participants in the occasional and daily groups, and across a range of doses. This decrease was only statistically significant in certain dose groups. This finding is excepted by the 10–20mg edible group at the post 2 timepoint (expected peak drug effect), where we observed a non-clinically significant (~ 5bpm) increase in HR.

**Discussion:**

This analysis suggests that edible cannabis does not have the same impact on HR as inhaled cannabis products. This analysis also suggests that commercially available edible cannabis does not produce a robust cardiovascular response when used naturalistically.

## Introduction and Background

Cannabis is easy to obtain and widely used in the United States; an estimated 74% of people live in a state where cannabis is legal, and 1 in 4 adults report using cannabis in the past year (1–2). Edible cannabis products are growing in popularity and currently account for an estimated 12.4% of legal cannabis sales (3). In Colorado, 47% of adults who currently use cannabis report consuming edible cannabis in the past 30 days (4).

Oral consumption of cannabis is accompanied by slow onset/extended duration of drug effects, and greater variability in absorption compared to cannabis inhalation (5–6). However, the pharmacodynamic and physiological differences between inhaled and edible cannabis remain relatively understudied. Most research that explores cardiovascular effects of tetrahydrocannabinol (THC) focuses on inhaled cannabis products and consistently demonstrates substantial increases in heart rate (HR) following acute use (6–8).

Fewer studies have examined the acute cardiovascular effects of edible cannabis. Among those that have, many use controlled dosing protocols, some of these involved edibles derived from federally sourced cannabis or dronabinol, a synthetic pharmaceutical cannabinoid (5, 7, 9). Prior studies have consistently reported a relationship between increase in heart rate and blood THC concentrations following acute oral THC administration (5; 10–14). Tachycardia is a commonly reported response, and limited evidence suggests that bradycardia may also occur following acute cannabis use (15).

With a few exceptions that use a naturalistic study design, prior studies may not generalize to commercially available edible cannabis products and contemporary patterns of use (6–7). The primary objective of this naturalistic within-person study was to explore how acute use of edible cannabis impacts heart rate.

## Materials and Methods

As part of a larger study of cannabis impairment, participants (N = 59) were recruited and consented into one of two groups based on self-reported frequency of their cannabis consumption. Participants who used any cannabis product daily (at least the last 29 of 30 days) or occasionally (at least one time/month, no more than three times/week), were classified as proxies for tolerance. The Colorado Multiple Institutional Review Board approved study procedures.

### Study Screening

Study participation consisted of two visits, first a brief (~ 30 minute) visit to obtain informed consent and screen for key eligibility criteria (e.g., healthy baseline BP/HR, urine drug test, pregnancy test, and questionnaires). If eligible, participants were invited back for a data collection visit.

### Baseline Data Collection

Participants were asked to abstain from cannabis use for12 hours before the start of their data collection visit. At the start of the visit participants self-reported significant medical history, medication use and cannabis use history. HR measurements were taken with a digital blood pressure monitor (Omron HEM-907XL). A resting HR at baseline above 100 beat per minute met criteria for study exclusion (16). At baseline, after baseline HR was collected, an intravenous catheter (IV) was placed and approximately 6mL of blood was collected.

### Cannabis Consumption

Participants were asked to bring their own cannabis edibles labeled with THC concentration, containing no more than a 1:1 ratio of cannabidiol (CBD) from a licensed Colorado dispensary. Details of the product label were recorded to obtain dose (e.g. milligrams THC). After baseline assessments participants consumed their cannabis edible. Participants’ cannabis use was not directed by staff; we asked participants if they usually consumed at least 5 mg of THC and we restricted edible types to tablets, capsules, gummies, or powders. After cannabis consumption participants were given a break and data collection resumed approximately 45 minutes later.

### Blood and HR timing and Medications

Venous blood samples were taken with an IV at baseline, and an average of 43 (post 1, P1), 89 (post 2, P2) and 178 minutes (post 3, P3) post cannabis consumption. Heart rate was collected at baseline, 39 (post 1, P1), 84 (post 2, P2), and 163 (post 3, P3) minutes post cannabis consumption. Medications and medical conditions that suppress heart rate or induce tachycardia were recorded.

### Statistical Analysis

Primary outcomes were HR and THC concentration in whole blood. Dose consumed by each participant was categorized into quartiles,. Each outcome was analyzed using a linear mixed effects model with fixed effects for dose category, timepoint, and the interaction between timepoint and dose category. The heart rate model also controlled for the use of medications known to affect heart rate. For all models, a subject-specific random intercept was included to account for within-participant repeated measures. Differences in least-squares means between baseline and post-consumption timepoints were calculated and assessed for each dose group. Pre- to post-period least-squares mean changes were also estimated within each group and compared across groups to assess the effect of dose on the two outcomes. Statistical significance was defined as p < 0.05. Analyses were conducted using R v4.5.

## Results

### Participant and Edible Dose Characteristics

Of the 59 participants in the study n = 29 used cannabis daily and n = 30 used cannabis occasionally. Doses range from 5mg to 100mg. Participants were organized into the following dose groups: <10 mg (n = 18), 10 mg (n = 19), > 10 mg and < 20 mg (n = 11), and ≥ 20 mg (n = 11). Additionally, n = 9 participants self-reported use of medications known to affect heart rate.

### HR Post Consumption

**Figure 1:**
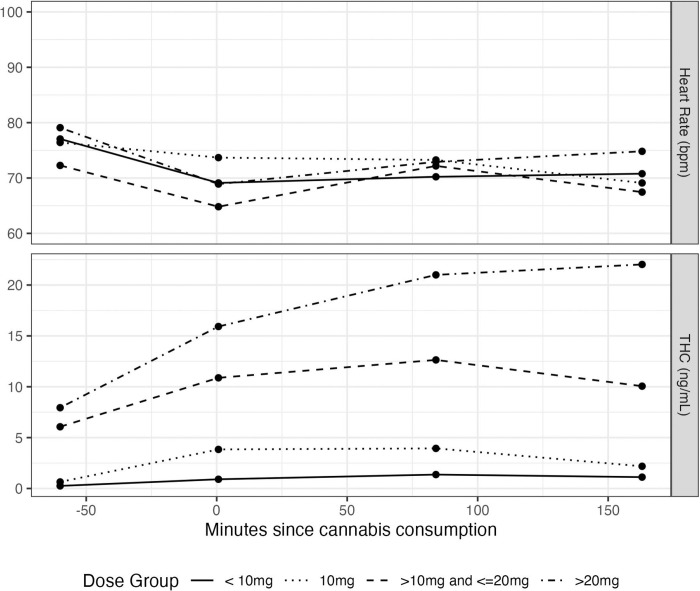
Change in Heart Rate and Blood THC Over Time by Dose of Cannabis Edible

Table 1 reports HR at four time points. Overall, we did not observe clinically significant increases in post-consumption HR, regardless of edible dose (17). In fact, when comparing the HR at 40 minutes post consumption to baseline, we found a pattern of a statistically significant decline in HR for three of the four dose groups At P1 and P2 (40 minutes and 84 minutes after consumption), there was a statistically significant decrease in HR for those using < 10mg of THC, from 78.0 BPM to 70.0 and 71.1, respectively. Similarly, we found a decrease in HR from baseline to P3 for those who took 10 mg (from 77.1 to 69.8 BPM) and from baseline to P1 for those taking > 20 mg (from 80.1 to 69.9 BPM) (Fig. 1).

### HR change by dose of edible cannabis

For participants who consumed less than 10 mg, average heart rate significantly decreased from pre-to P1, P2, and P3 (all p < 0.01) (Table 1). For those who consumed 10 mg, heart rate decreased by an average of 7.3 BPM from the pre-to post 3 (p < 0.01). For participants who consumed more than 20 mg, average heart rate significantly decreased from pre-to P1 (p < 0.01) and post 2 (p = 0.04) timepoints (Fig. 1).

### Blood THC by dose

**Figure 2:**
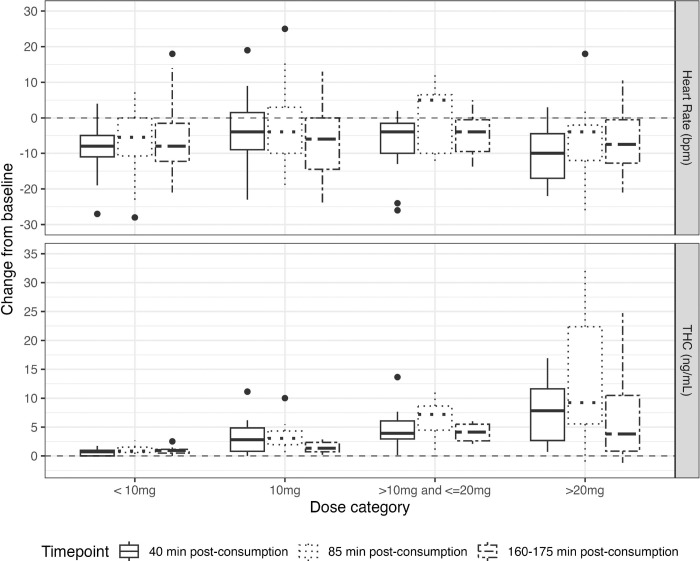
Within Participant change in Heart Rate and Blood THC from Baseline to Post-Consumption by Dose of Cannabis Edible. Note: One observation was not included in this figure, but was included in analysis, because it was an outlier that visually compressed the data.

Table 1 reports delta-9-THC in whole blood for each timepoint. Blood THC did not significantly increase from pre- to any post timepoints for participants who consumed 10mg or less. In the > 10 mg-20 mg group, average THC levels significantly increased from pre-to P1 (p = 0.048) and P2 (p < 0.01) timepoints. For participants who consumed more than 20 mg, average THC levels were significantly higher at all post-consumption timepoints compared to the pre-consumption timepoint (all p < 0.01) (Table 1, Fig. 2)

### Note

One observation was not included in this figure, but was included in analysis, because it was an outlier that visually compressed the data.

## Discussion

This study examined changes in HR and blood THC following edible cannabis consumption among participants with varying cannabis tolerance who used a wide range of self-selected doses of edible cannabis products that are available on the legal market. Overall, we observed a non-clinically significant decrease in HR in the hours after use, regardless of dose. Simultaneously, there was an overall increase in THC in the blood. This is a departure from some prior studies which more consistently demonstrate an increase in HR post use, and the demonstrated relationship between an increase in HR and THC in the blood (5, 10–14).

The small decrease in HR could be due to several factors. Participants were able to dose themselves; it is unlikely they elected to take doses that would make them physically uncomfortable, which can happen in studies where participants with little tolerance take high doses such as 20 mg (9). Over half of study participants (n = 33) reported that they use edibles primarily for sleep or relaxation before bed, with others reporting use for pain management and taking a break from inhaling cannabis. This could mean that people had expectations the effects of edible cannabis would be calming or sedative, and physiological effects are mirroring this expectation. Many participants also inhale cannabis as part of their typical use. It is possible that our results are indicative of tolerance to mode versus tolerance based on frequency of use, which has been studied in the context of subjective drug effect (18).

A limitation of the study is that we did not use an experimental design, so tolerance and dose consumed are inherently dependent. However, this is a complexity even in experimental designs; they may fail to include the full range of participants, inclusive of those with tolerance, or they may fail to determine the appropriate dose. Additionally, participants used products purchased from a dispensary rather than those created by a research pharmacist. However, our prior work suggests that edible cannabis in Colorado is reasonably accurately labeled adding validity to this study (19).

Our study enhances the existing literature on cardiovascular response to edible cannabis with a naturalistic approach and typical use of commercially available edible cannabis. Future studies should explore why there might be a different cardiovascular response between inhaled and edible cannabis (15).

## Conclusion

Our results suggest that edible cannabis, when used naturalistically, might not produce a robust cardiovascular response.

## Figures and Tables

**Table 1:** Blood THC Metabolites: Adjusted Models Comparing pre-post changes in Heart Rate and THC by Cannabis Edible Dose Note.

*Outcome*	*Dose Consumed*	*N*	*Baseline*	*Post 1 (~40 minutes)*		*Post 2 (~85 minutes)*		*Post 3 (~160–175 minutes)*	
			Mean	Mean	p-value	Mean	p-value	Mean	p-value
HR	< 10mg	18	78.0	70.0	**<0.01**	71.1	**<0.01**	71.7	**<0.01**
HR	10mg	19	77.1	74.4	0.22	74.0	0.16	69.8	**<0.01**
HR	>10mg to <=20mg	11	73.0	65.6	**0.01**	72.9	0.98	68.2	0.10
HR	>20mg	11	80.1	69.9	**<0.01**	73.9	**0.04**	75.8	0.14
THC	< 10mg	18	0.3	0.9	0.73	1.4	0.56	1.1	0.65
THC	10mg	19	0.6	3.8	0.08	3.9	0.08	2.9	0.40
THC	>10mg to <=20mg	11	6.1	10.9	**0.048**	12.6	**<0.01**	10.1	0.10
THC	>20mg	11	7.9	15.9	**<0.01**	21.0	**<0.01**	22.0	**<0.01**

HR = heart rate

## Data Availability

The de-identified datasets used and/or analyzed during the current study are available from the corresponding author on reasonable request.
